# Automated Speech Analysis in Bipolar Disorder: The CALIBER Study

**DOI:** 10.1192/j.eurpsy.2025.1043

**Published:** 2025-08-26

**Authors:** G. Anmella, M. De Prisco, J. B. Joyce, C. Valenzuela-Pascual, G. Chatzisofroniou, D. Hidalgo-Mazzei, E. Vieta

**Affiliations:** 1 Bipolar and Depressive Disorders Unit, Hospital Clinic of Barcelona, Barcelona, Spain; 2 School of Graduate Medical Education, Mayo Clinic, Rochester, United States

## Abstract

**Introduction:**

**Background:** Bipolar disorder (BD) is marked by dramatic mood and energy shifts, often mirrored in speech. Traditional diagnosis and monitoring largely rely on subjective clinical assessments. However, advancements in natural language processing (NLP) present an opportunity for more objective speech pattern analysis.

**Objectives:**

**Aims:** This study aims to (i) identify correlations between speech features and BD symptom severity, (ii) create predictive models for diagnostic and treatment outcomes, and (iii) pinpoint significant speech features and optimal tasks for analysis.

**Methods:**

The CALIBER study is a longitudinal, observational project collecting audio from BD patients during euthymia, acute manic or depressive episodes, and recovery phases. Patients engaged in clinical interviews, cognitive assessments, standard readings, and storytelling (**Figure 1**). Automatic diarization and transcription enabled the extraction of speech features, including acoustic properties, linguistic content, formality, and emotionality. Analyses include (i) correlation of speech features with clinical scales, (ii) predictive modeling using lasso logistic regression, and (iii) feature importance identification.

**Results:**

Preliminary data from 76 patients (24 manic, 21 depressed, 31 euthymic) were analyzed. The cohort had a mean age of 46.0 ± 14.4 years, 63.2% female. Mean YMRS scores dropped from 22.9 ± 7.1 to 5.3 ± 5.3 post-mania, and HDRS-17 scores in depressed patients fell from 17.1 ± 4.4 to 3.3 ± 2.8 post-recovery. Euthymic patients showed lower baseline scores.

**Conclusions:**

Automated speech analysis in BD can provide objective biomarkers for diagnosis and monitoring, highlighting subtle pre-relapse changes and informing treatment strategies. Establishing standardized protocols is vital for developing a global speech database to support collaborative research and enhance BD management.

**Disclosure of Interest:**

G. Anmella Grant / Research support from: GA has received CME-related honoraria, or consulting fees from Adamed, Angelini, Casen Recordati, Janssen-Cilag, Lundbeck, Lundbeck/Otsuka, Rovi, and Viatris, with no financial or other relationship relevant to the subject of this article., Consultant of: GA has received CME-related honoraria, or consulting fees from Adamed, Angelini, Casen Recordati, Janssen-Cilag, Lundbeck, Lundbeck/Otsuka, Rovi, and Viatris, with no financial or other relationship relevant to the subject of this article., M. De Prisco: None Declared, J. Joyce: None Declared, C. Valenzuela-Pascual: None Declared, G. Chatzisofroniou: None Declared, D. Hidalgo-Mazzei: None Declared, E. Vieta: None Declared

